# Activity-induced MEMRI cannot detect functional brain anomalies in the APPxPS1-Ki mouse model of Alzheimer’s disease

**DOI:** 10.1038/s41598-018-37980-y

**Published:** 2019-02-04

**Authors:** Alexandre Androuin, Yah-se Abada, Myriam Ly, Mathieu Santin, Alexandra Petiet, Stéphane Epelbaum, Anne Bertrand, Benoît Delatour

**Affiliations:** 1 Institut du Cerveau et de la Moelle épinière, INSERM U1127, CNRS UMR7225, Université Pierre et Marie Curie, Sorbonne Universités, Paris, France; 2 Center for Neuroimaging Research, Institut du Cerveau et de la Moelle épinière, Paris, France; 3 Centre des Maladies Cognitives et Comportementales, Sorbonne Universités, Hôpital de la Salpêtrière, Paris, France; 4 Aramis Project Team, Inria Research Center of Paris, Paris, France; 5 Institut Roche, Boulogne-Billancourt, France

## Abstract

Alzheimer’s disease (AD) is the most common cause of dementia. Aside neuropathological lesions, abnormal neuronal activity and brain metabolism are part of the core symptoms of the disease. Activity-induced Manganese-Enhanced Magnetic Resonance Imaging (MEMRI) has been proposed as a powerful approach to visualize evoked brain activity in rodents. Here, we evaluated the relevance of MEMRI in measuring neuronal (dys-)function in the APPxPS1 knocked-in (KI) mouse model of AD. Brain anomalies were firstly demonstrated in APPxPS1-Ki mice using cognitive testing (memory impairment) and histological mapping of immediate early gene products (decreased density of fos-positive neurons). Paradoxically, MEMRI analyses were not able to confirm the occurrence of neuronal hypoactivities *in vivo*. We then performed a neuropathological analysis that highlighted an abnormal increased permeability of the blood-brain barrier (BBB) in APPxPS1-Ki mice. We hypothesized that diffuse weakening of the BBB results in an uncontrolled diffusion of the MR contrast agent and a lack of correlation between manganese accumulation and neuronal activity. These results bring to light a limitation of the activity-induced MEMRI approach when applied to the APPxPS1-Ki mouse model as well as other mouse models harboring a compromised BBB.

## Introduction

The onset of clinical symptoms in Alzheimer’s disease (AD) is paralleled and even preceded by functional brain changes as a response to the insidious and progressive development of neuropathological lesions over the course of decades^1^.

Being able to monitor brain activity throughout disease progression and in response to targeted therapies is fundamental to refining diagnosis in patients and assessing the effectiveness of symptomatic or disease-modifying interventions. Functional imaging is routinely performed in humans, yet *in vivo* and non-invasive imaging methods for preclinical (animal) models of the disease have not been investigated to the same degree. In the APPxPS1-Ki mouse model of AD, which displays both brain amyloidosis and neuronal loss, we previously found evidence of brain perfusion anomalies in 6-month-old animals using arterial spin labelling (ASL)^2^. However, this method suffers from poor spatial resolution. Similarly, positon emission tomography (PET) is limited in rodents due to its low resolution on the order of millimeters.

Manganese-Enhanced Magnetic Resonance Imaging (MEMRI) has been developed in rodents from the pioneering work of Koretsky^3^ as a technique to enhance the visualization of the brain’s architecture, perform fiber tracing, and also visualize brain activity *in vivo*. Divalent manganese ions (Mn2+) are paramagnetic and cause a reduction in T_1_ relaxation time. Mn2+ can enter excitable cells through voltage gated Ca2+ channels and the Na+/Ca2+ exchanger. Enhanced neuronal activity therefore increases intraneuronal Mn2+ concentrations which in turn allow for spatial localization of discrete neuronal activities with MRI. Intraneuronal Mn2+ accumulation has been correlated with other signatures of neuronal activity provided by calcium imaging^4^ or electrophysiological recordings^5^. In addition, brain activity, as mapped with MEMRI, is accompanied by hemodynamic changes (blood-oxygen-level dependent contrast imaging, cerebral blood flow) and immediate early gene products induced by neuronal activity^6–9^.

Although not applicable to human studies due to the known toxicity of manganese^10^, MEMRI has been applied for mapping brain activity in rodents not only in response to sensory and motor stimulations^11–15^ but also more complex and integrated contexts such as drug administration/withdrawal^16,17^, feeding/fasting^18,19^, exploration of novel environments^20^, and learning & memory paradigms^21^. More recently, MEMRI-based investigations have been performed to evaluate functional brain anomalies in mouse models of AD^20,22–24^.

In the present study, we performed activity-induced MEMRI (AIM) in APPxPS1-Ki mice to evaluate anomalies of brain activity in this AD model *in vivo* and at high resolution. Furthermore, we benchmarked MEMRI assessments with macroscopic evaluations of cognitive function and a standard analysis of neuronal activity (histological mapping of immediate early genes products).

## Results

### Decreased neuronal activity as measured by fos mapping and spatial memory impairments in APPxPS1-Ki mice

**Figure 1:**
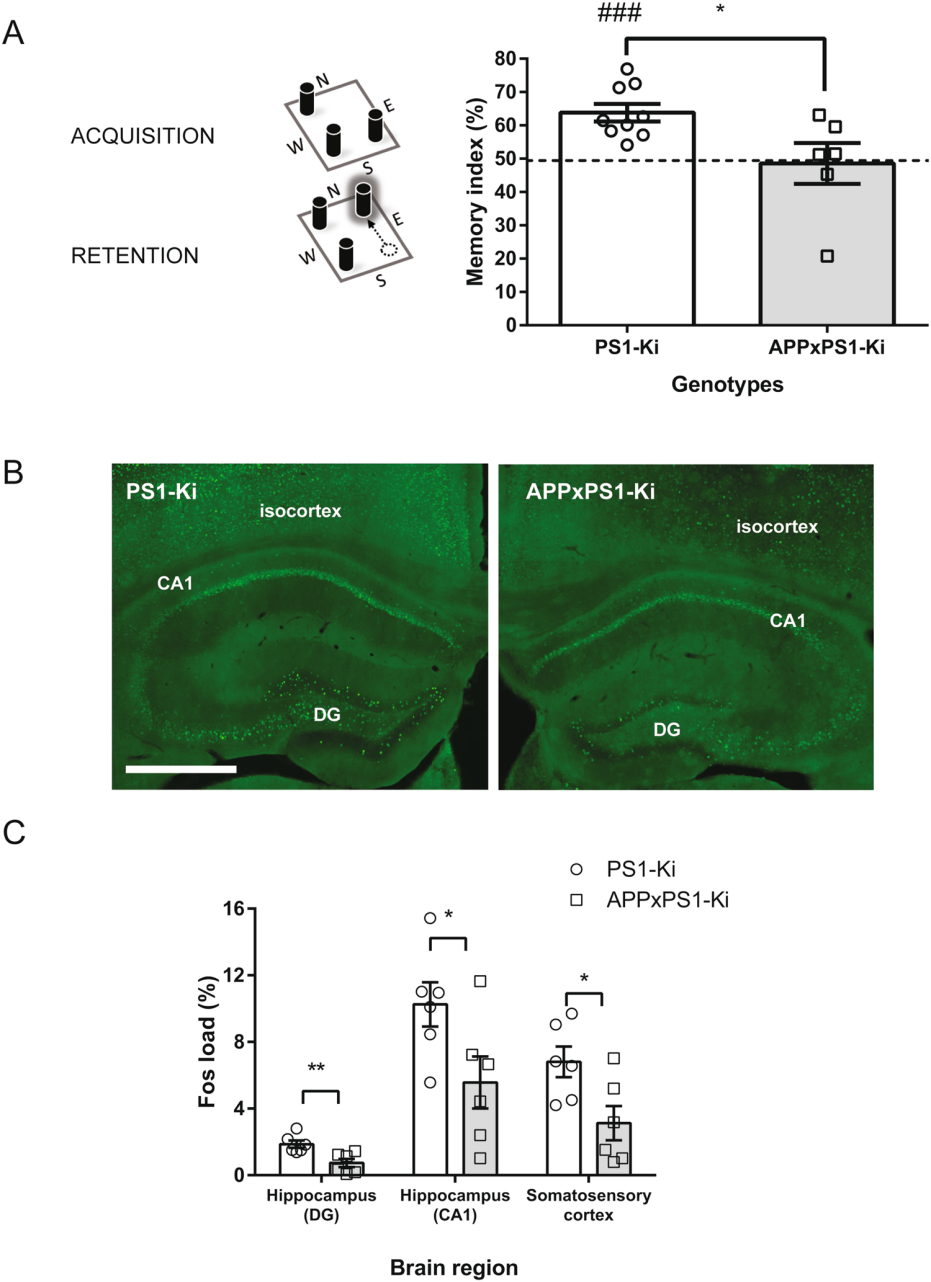
Impaired spatial cognition and reduced brain activity in APPxPS1-Ki mice. (**A**) The general protocol of the object recognition task is schematized on the left. Mice were first trained with a configuration of three identical objects (ACQUISITION phase) and then retested after a spatial change occurred in the arena (RETENTION phase). Good memory performance is assessed by a detection of the spatial change (i.e., increased exploration of the displaced object). The right part of the figure illustrates the good memory score of PS1-Ki mice that performed well above chance level (dotted line) while APPxPS1-Ki mice were significantly impaired. *p < 0.05. ^###^p < 0.001 (different from chance level). (**B**) Representative microphotographs of fos immunoreactivity in PS1-Ki and APPxPS1-Ki mice after behavioral stimulation. Note the decrease in the number of fos-positive nuclei in the isocortex and hippocampal subfields of APPxPS1-Ki mice. DG: dentate gyrus. Scale bar: 500 µm. (**C**) Quantification of fos immunostaining in three different regions of interest. For all regions, APPxPS1-Ki mice displayed decreased immunoreactivity levels. *p < 0.05; **p < 0.01.

The first step in assessing brain function in young (4–6 month-old) APPxPS1-Ki mice was with a cognitive evaluation. Mice were trained in a standard recognition memory paradigm (Fig. 1A). They were placed in a new environment and allowed to explore a particular configuration of objects. Then after a short retention interval, mice were tested for their ability to detect a spatial change in the objects’ configuration. Spatial recognition primarily requires an intact hippocampus^25^, a brain region that undergoes gradual, age-dependent neurodegeneration in APPxPS1-Ki mice^26^. PS1-Ki mice (that display no specific neuropathological alterations, behave like wild-type mice and can be used as control animals^2^) were able to detect the spatial change in relation to the initial memorized scene and showed an exploration bias towards the displaced object (t(8) = 5.274, p < 0.001). On the other hand, APPxPS1-Ki age-matched littermates were unable to show differential object exploration above chance (t(5) = 0.234, ns) and presented with a lower memory score in comparison to PS1-Ki mice (t(13) = 2.588, p < 0.025; Fig. 1A).

We then investigated neuronal dysfunction as a substratum for underlying cognitive dysfunction in APPxPS1-Ki mice. We mapped immediate early gene products to assess levels of activity-dependent neuronal activation^27,28^. Using this post-mortem approach, which required quantitative immunohistochemical analysis of the fos protein after a behavioral stimulation (here, sequential exploration of two stimuli-enriched open fields for a total duration of two hours), we observed a reduced number of activated neurons in the brain of APPxPS1-Ki mice when compared to PS1-Ki littermates. APPxPS1-Ki mice displayed an overall pattern of neuronal hypoactivity that was present in hippocampal subfields (dentate gyrus and CA1 pyramidal cell layer; Fig. 1B) but also in other brain regions such as the somatosensory cortex (Fig. 1C). Statistical analysis confirmed the decreased activity-associated fos immunoreactivity in all sampled brain areas (ts(10) > 2.66; ps < 0.05).

In summary, both behavioral and fos mapping data highlighted a clear brain dysfunction associated with neuronal hypoactivation in APPxPS1-Ki mice. These findings extend our previous behavioral and imaging data^2^.

### Paradoxical normal brain activity in APPxPS1-Ki mice as detected by *in vivo* imaging using activity-induced MEMRI

We then evaluated the potential of MRI-based methods (activity-induced MEMRI -AIM) in detecting and quantifying brain hypoactivity in APPxPS1-Ki mice. We made use of a published protocol^20^ that had been previously applied to characterize neuronal dysfunction in a tau transgenic mouse model. In summary, animals were first injected with the contrast agent. They then were behaviorally stimulated with a protocol similar to that used for the aforementioned fos-mapping experiments. Finally, mice underwent an MRI examination to map levels of manganese intake as a correlate of local neuronal activation.

We first performed a series of pilot experiments in wild-type mice to validate the protocol (data not shown). These pilot experiments confirmed that our overall procedure was adequate to detect significant T_1_ decreases in brain tissue following MnCl2 injection and behavioral stimulation. This T_1_ decrease was not observed in MnCl2-injected mice that did not receive a behavioral stimulation, underlining that manganese accumulation was activity-dependent. Moreover, the behavioral stimulation in mice injected with saline was not sufficient to promote a decrease in T_1_, indicating that the MnCl2-behavioral stimulation pairing was required for manganese brain accumulation to map evoked neuronal activity.

**Figure 2:**
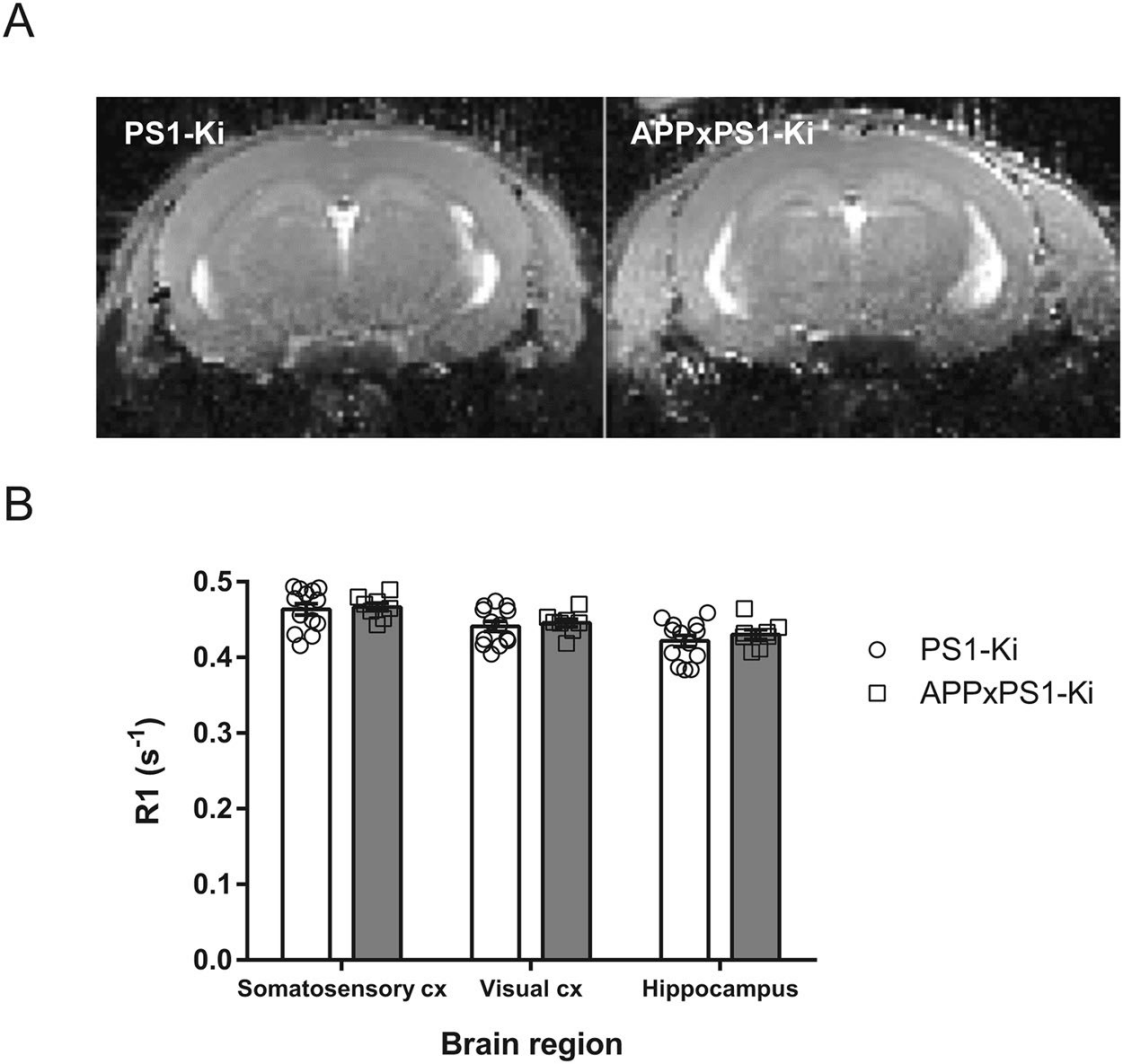
Activity-induced MEMRI in APPxPS1-Ki mice. (**A**) Representative illustration of T_1_-weighted images of mouse brains (coronal plane) after MnCl2 peripheral injection and behavioral stimulation. No obvious differences are observed between PS1-Ki and APPxPS1-Ki genotypes in terms of MR contrast. (**B**) Quantification of R1 relaxivity in different regions of interest in mice that underwent activity-induced MEMRI. Regardless of the brain region, no differences were obtained between PS1-Ki and APPxPS1-Ki mice.

A large cohort of transgenic mice was then used for the main AIM experiment. Contrary to our expectations, we did not detect any differences in signal intensity between APPxPS1-Ki and PS1-Ki mice after behavioral stimulation (Fig. 2A). Relaxation rates were similar in the two genotypes not only in the hippocampus, which is classically activated during spatial encoding of new environments, but also in cortices activated by somatosensory and visual cues provided during the behavioral stimulation epoch (all ts(19) < 0.778; ns; Fig. 2B).

Overall, on the sole basis of activity-induced MEMRI, we were unexpectedly unable to detect differences in brain activations between APPxPS1-Ki and PS1-Ki controls.

### Integrity of the blood-brain barrier of APPxPS1-Ki mice

We explored the integrity of the BBB in APPxPS1-Ki mice with the underlying hypothesis that a compromised barrier might be the source of uncontrolled brain seepage of manganese in this mouse model.

**Figure 3:**
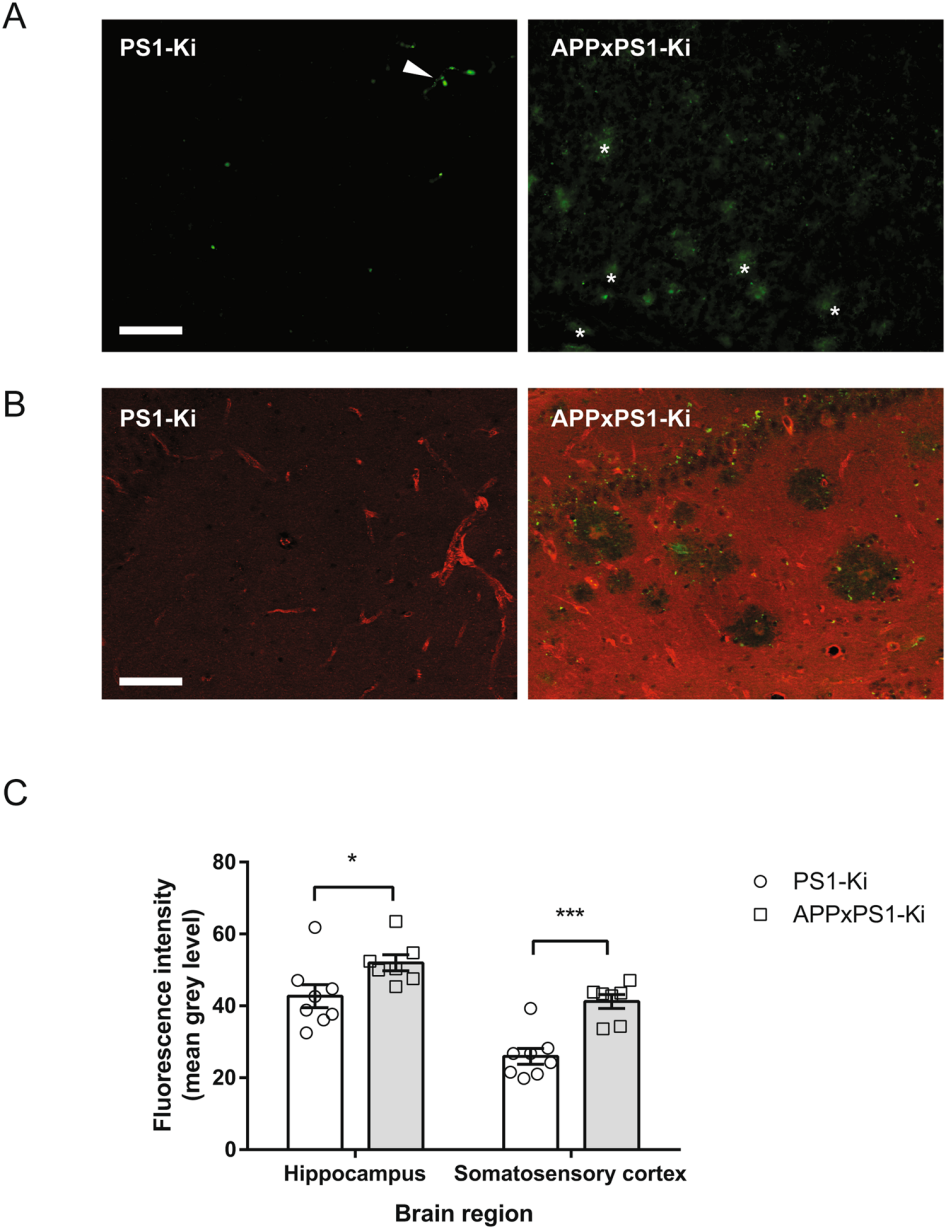
Blood brain barrier permeability in APPxPS1-Ki mice. (**A**) Immunodetection of mouse immunoglobulins in the hippocampus. Note the faint signal in PS1-Ki mice limited to blood vessels (white arrow heads) and absent from brain parenchyma. On the other hand, a diffuse immunoreactivity was observed in the hippocampus of APPxPS1-Ki mice and was reinforced around plaques (examples of IgG-positive plaques pointed with white asterisks). (**B**) Immunodetection of Aß deposits (green) and albumin (red). In PS1-Ki mice, albumin was only detected in the lumen of blood vessels, and no Aß immunoreactivity was observed. On the other hand, albumin was diffusely deposited in the brain of APPxPS1-Ki mice and accumulated in the core of amyloid deposits but not in its immediate periphery. Scale bar for A and B: 50 µm. (**C**) Quantification of albumin immunoreactivity in the hippocampus and somatosensory cortex. In both regions, increased immunoreactivity was observed in APPxPS1-Ki mice as compared to PS1-Ki littermates. *p < 0.05; ***p < 0.001.

To evaluate BBB permeability, we assessed the presence of serum proteins in the brain parenchyma with immunohistochemistry. In control PS1-Ki mice, we observed that resident immunoglobulins and albumin proteins were strictly confined to the lumen of blood vessels and showed no visible extravasation in surrounding brain tissue (Fig. 3A,B). On the other hand, in APPxPS1-Ki mice, both types of serum proteins had a vascular localization but were also abundantly present within the brain parenchyma. Strong immunoreactivity was observed at the vicinity of amyloid deposits replicating observations in other AD mice models showing anomalies of the BBB^29^. No focal hemorrhages were observed in APPxPS1-Ki mice. However, a quantitative analysis confirmed a higher level of serum protein (albumin) in different brain regions of APPxPS1-Ki mice (comparison PS1-Ki mice vs APPxPS1-Ki mice: ts(13) > 2.304; ps < 0.05; Fig. 3C).

These data provide evidence of a diffuse leakage of the BBB in APPxPS1-Ki mice.

## Discussion

Neuronal dysfunction is the early and defining pathology underlying cognitive decline in AD. In the present work, we evidenced that APPxPS1-Ki mice that display an aggressive cerebral amyloidosis also develop brain dysfunction (impaired recognition memory) associated with neuronal hypoactivity in different brain regions as evidenced by fos mapping. These observations are supported by previous data from the literature on the model as we and others showed that APPxPS1-Ki are behaviorally impaired^2,30,31^ and display brain hypoperfusion^2^.

Our principal goal was to implement an *in vivo* high-resolution MRI-based methodology to further investigate neuronal dysfunction in this preclinical model. However, contrary to our expectations, MEMRI analysis was not able to show any differences in brain activity levels between controls and APPxPS1-Ki mice. We hypothesized that this paradoxical result (1) points to a limitation in the application of activity-induced MEMRI and (2) is related to non-specific manganese uptake that impedes the measurement of specific functional activation. Manganese diffusion in the brain parenchyma is indeed known to be dependent on BBB integrity. One of the first brain regions to show a T_1_ decrease after manganese injection is the pituitary gland, presumably due to the local BBB permeability^19,32^. In brains from AD patients and from numerous mouse models with amyloid plaques or neurofibrillary tangles, evidence of BBB leakages has been reported (for review^33^). We also collected congruent histological observations and quantifications highlighting a diffuse BBB permeability in APPxPS1-Ki that may allow for a uncontrolled diffusion of manganese and a lack of correlation between the accumulation of the MR contrast agent in discrete brain regions and local neuronal activity. It therefore appears crucial to investigate BBB permeability in mouse models developing neurodegenerative phenotypes before drawing firm conclusions from MEMRI data. Retrospectively, one may question whether previous reports showing increased manganese intake in AD transgenics^22,24^ reliably revealed abnormal patterns of neuronal activity or, alternatively, underlined a non-specific accumulation of manganese due to a weakened BBB. MEMRI has been developed as a very attractive method to map brain activity at high resolution^34^, is minimally invasive, and relies directly on calcium influx rather than on indirect hemodynamics-based functional MRI methods. In the present work, MEMRI was nevertheless unable to identify brain dysfunction in an aggressive AD mouse model despite clear functional anomalies in the same animals by three concurrent methods (ASL, fos mapping, cognitive testing). Our data, combined with the known toxicity of manganese, therefore draw attention to possible limits and biases in the interpretation of MEMRI data due to uncontrolled leakage of manganese in specific circumstances.

## Methods

### Animals

The APPxPS1-Ki mouse model has been previously described^26^. It harbors the M233T & L235P knocked-in endogenous mouse PS1 gene and overexpresses Swedish K670N/M671L and London V717I APP mutations under the control of a Thy1 promoter. The APPxPS1-Ki line develops an early-onset and aggressive neuropathological phenotype, including the formation of intracellular inclusions, extracellular amyloid deposits^26^, and Aß oligomer accumulation^35^. APPxPS1-Ki male mice between 4 and 6 months-old were used for the present study. PS1-Ki aged-matched littermates were used as controls as these mice displayed no brain lesions and no altered behavioral/cognitive phenotypes^2^. The general health of mice was regularly checked and body weights were assessed weekly throughout the experimental period. All experiments on animals were conducted in accordance with the ethical standards of French and European laws (European Communities Council Directive of 24 November 1986). The supervisor of the present study (B. D.) has received official approval from the French Ministry of Agriculture to carry out research and experiments on animals (Authorization No. A-75-1741). The project was approved by the local ethics Committee (Charles Darwin Committee; Project No. 643.02).

### Novel object location task

Nine PS1-Ki mice and six APPxPS1-Ki mice were used for this experiment.

The novel object location test is based on the natural tendency of rodents to investigate a displaced object. Our protocol was adapted from a previously described procedure^36^. Before test initiation, animals were habituated to the experimenters (2–5 min handling/mouse/day during DAY 1 and DAY 2). The behavioral test was carried out in a dimly illuminated (8.5 lux) square gray PVC open-field box (50 cm × 50 cm × 30 cm) with white masking noise (64 dB). During DAY 3 and DAY 4, mice were given a familiarization session of 15 min in which they were allowed to freely explore the empty open-field arena. The next day (DAY 5), 3 identical objects were introduced in the arena at South-West, North-West and South-East locations in the squared arena (Fig. 1A) and mice had a free-exploration session for 10 min. On DAY 6, the proper learning & memory test was carried out: mice were first exposed to a triplet of identical objects (different from the ones used on DAY 5 but located with the same geometrical configuration) for two consecutive 10 minutes training sessions separated by 5 minutes during which animals were returned to their home cages. Five minutes after the last training session, the mice were replaced in the arena with the same objects and similar spatial configuration except that one object was moved from its original location to the unused North-East position (Fig. 1A). The retention test ended after 10 min of free exploration. Recognition memory relying on detection of the spatial change between training and retention phases was evaluated by measuring the exploration bias towards the displaced object. Exploration was defined as the presence of the mouse nose in the 1 cm annulus surrounding objects. This measure was automatically obtained thanks to a videotracking system (ANY-maze, Stoelting, USA). A memory index was calculated as the percent of time exploring the displaced object over the mean time exploring the 2 non-displaced objects and evaluated using a one-sample Student t-test. Difference between genotypes was assessed using an unpaired t-test.

### Activity-induced fos mapping

Six PS1-Ki mice and six APPxPS1-Ki mice were used for this experiment.

A behavioral-stimulation paradigm was applied to map neuronal activity. The animals were successively exposed to two “stimulating” environments in 50 × 50 × 30 cm square open fields containing various junk objects, tunnels on the floor and visual cues placed on the walls. Light was setup to 15 lux, and external speakers diffused 60 dB ambient music during the exploration period, which lasted 2 hours (one hour in each open field) and that associated multisensory and motor stimuli. Mice were then euthanized with an overdose of sodium pentobarbital and perfused transcardially with a 4% buffered formaldehyde solution. After cryoprotection, brains were sectioned on a freezing microtome.

One series of sections was processed to perform double immunofluorescent staining of fos and NeuN proteins.

After blocking non-specific antigenic sites with 3% normal donkey serum and 3% bovine serum albumin, sections were incubated overnight at RT with a cocktail of mouse anti-NeuN (Chemicon-Millipore MAB377; dilution 1:2500) and rabbit polyclonal anti-fos (AB-5, Calbiochem-VWR, France; dilution 1:5000) primary antibodies. Sections were then incubated for 2 h in Alexa Fluor 488-conjugated donkey anti-rabbit and Alexa Fluor 555-conjugated donkey anti-mouse IgG secondary antibodies (Invitrogen-ThermoFisher scientific, France; dilution 1:250). Sections were mounted on glass slides and allowed to dry. Finally, sections were incubated for 1 min with DAPI and coverslipped with Mowiol mounting medium.

Immunoreactivity was quantified using the ICY program developed by the Quantitative Image Analysis Unit at Pasteur Institut (http://www.bioimageanalysis.org). This freeware automatically calculated the proportion of fos stained tissue (p = stained fos area/stained NeuN area), providing unbiased stereological measurements of the “fos load”. The surface occupied by all neurons (NeuN+ cells) was segmented in the red channel using the Otsu thresholding method while the surface of fos-positive neurons was automatically thresholded in the green channel after background subtraction. Three Regions Of Interest (ROIs) were analyzed: somatosensory cortex, dentate gyrus and CA1 field of the hippocampus. For each ROI, measurements were performed on three different sections. Results were then averaged to give a reliable quantitative evaluation of local fos immunostaining. Comparisons between groups were performed using t-tests.

### Activity-induced MEMRI (AIM)

Thirteen PS1-Ki mice and eight APPxPS1-Ki mice were used for this experiment. The first published AIM studies were performed in conjunction with BBB disruption to enhance passage of manganese. This paradigm, however, precludes the study of awake and freely-moving animals outside the magnet. Yu and collaborators subsequently demonstrated that the BBB did not need to be weakened in order to measure neuronal function with MEMRI^37^, and we selected the latter approach to evaluate brain activity in APPxPS1-Ki in an ethological way (neuronal activity in response to the exploration of new environments).

The whole protocol was adapted from a previous study report^20^. We designed pilot experiments in C57BL/6 mice to optimize a protocol allowing for MRI detection of manganese chloride entry and diffusion in activated brain regions in behaviorally stimulated mice (data not shown).

Our protocol was then applied to APPxPS1-Ki and PS1-Ki mice: All mice received an intraperitoneal injection of 12.5 mM manganese chloride (MnCl2; 100 µM/kg) and were placed in their home cage for one hour. Preliminary experiments indicated that this dosage did not modify the general activity and body temperature of mice. Mice received the same two-hour behavioral stimulation (exploration of novel environments) as described for fos-mapping experiments (see above) and were then returned to their home cage for two hours before being analyzed by MRI.

A 11.7T Biospec spectrometer (Bruker, Germany) running the Paravision 5.1 MRI software was used for this study. A 72 mm birdcage emitter (Bruker) provided a radiofrequency emission, and a surface coil was used for signal reception (Bruker). During MRI, mice were anesthetized with isoflurane (1–2%) and a mixture of air and oxygen (1:5). Animals had their heads fixed with tooth and ear bars, their respiration rate was controlled over the whole experiment and the body temperature was maintained around 37 °C with circulating water.

To monitor the penetration of manganese into the brain parenchyma, R1 relaxation maps were acquired in every mouse. Indeed, R1 relaxation rates are linearly proportional to the underlying tissue Mn concentration and reflect the total absolute amount of Mn present in the tissue^38^. To acquire R1 maps, a Multi Slice 2D Spin Echo Sequence with varying repetition time (TR) was acquired. Parameters were: Matrix size = 96 ∗ 96, Field of View = 14.4 ∗ 14.4 mm² leading to an in-plane resolution of 150 µm isotropic, 16 slices were acquired with a slice thickness of 400 µm, Spectral Width = 100 KHz. Echo time (TE) was fixed to 11.94 ms, and TR was successively fixed to 304, 400, 500, 1000, 3000, 6000, and 9000 ms. Total scan time for R1 estimation was on the order of 32 min. Then, T1 parameter estimation was obtained using the fitting routine of the MRI software. Parametric images were inverted to obtain R1 parametric maps. ROIs were then manually defined using ImageJ software.

Comparison of R1 measures between groups was performed using unpaired t-tests.

### Histological analysis

Eight PS1-Ki mice and seven APPxPS1-Ki mice, randomly selected after the MEMRI experiment, were used for histological analysis aimed at evaluating the integrity of the blood-brain barrier. Animals were euthanized and perfused, and their brains were processed as described above (§ Activity-induced fos mapping).

For simple labeling of mice immunoglobulins, sections were incubated with an anti-mouse IgG conjugated to an Alexa 488 (Life Technologies, Saint-Aubin, France, 1:200, 2 hours at RT). Sections were then mounted on glass slides, dried, and mounted with Mowiol.

For double labeling of amyloid deposits and albumin, sections were incubated overnight at RT with a cocktail of antibodies, namely a polyclonal anti-albumin antibody (Dako, Glostrup, Danemark, 1:3000) and the biotinylated 4G8 anti-Aß antibody (Eurogentec, Agers, France, 1:5000). Sections were then incubated for 2 hours with secondary reagents: Alexa 455 goat anti-rabbit (Life Technologies, 1:1000) and Dylight 488 labeled streptavidine (Eurobio Abcys, Courtaboeuf, France, 1:500) before being mounted with Mowiol.

Albumin labeling was quantified by measuring mean grey levels with ImageJ^39^ from microphotographs acquired with an epifluorescence microscope. For each animal, a total of eight sections spanning the two ROIs (hippocampus and somato-sensory cortex) were analyzed to calculate a mean gray level reflecting staining intensity. Comparisons between genotypes were performed using unpaired t-tests.

## Data Availability

The datasets generated during and/or analyzed during the current study are available from the corresponding author on reasonable request.

## References

[1] Gordon BA (2018). Spatial patterns of neuroimaging biomarker change in individuals from families with autosomal dominant Alzheimer’s disease: a longitudinal study. The Lancet. Neurology.

[2] Faure, A. *et al*. Impaired neurogenesis, neuronal loss, and brain functional deficits in the APPxPS1-Ki mouse model of Alzheimer’s disease. *Neurobiol*. *Aging* **32** (2011). 10.1016/j.neurobiolaging.2009.03.009 19398247

[3] Lin YJ (1997). & Koretsky, a. P. Manganese ion enhances T1-weighted MRI during brain activation: an approach to direct imaging of brain function. Magn. Reson. Med..

[4] Kikuta S (2015). Quantitative activation-induced manganese-enhanced MRI reveals severity of Parkinson’s disease in mice. Sci. Rep..

[5] Svehla P, Bédécarrats A, Jahn C, Nargeot R, Ciobanu L (2018). Intracellular manganese enhanced MRI signals reflect the frequency of action potentials in Aplysia neurons. J. Neurosci. Methods.

[6] Aoki I, Naruse S, Tanaka C (2004). Manganese-enhanced magnetic resonance imaging (MEMRI) of brain activity and applications to early detection of brain ischemia. NMR Biomed..

[7] Duong TQ, Silva AC, Lee SP, Kim SG (2000). Functional MRI of calcium-dependent synaptic activity: cross correlation with CBF and BOLD measurements. Magn. Reson. Med..

[8] Hsu Y-H, Lee W-T, Chang C (2007). Multiparametric MRI evaluation of kainic acid-induced neuronal activation in rat hippocampus. Brain.

[9] Laine MA (2017). Brain activation induced by chronic psychosocial stress in mice. Sci. Rep..

[10] Dobson AW, Erikson KM, Aschner M (2004). Manganese neurotoxicity. Ann. N. Y. Acad. Sci..

[11] Aoki I (2002). Dynamic activity-induced manganese-dependent contrast magnetic resonance imaging (DAIM MRI). Magn. Reson. Med..

[12] Chen W, Tenney J, Kulkarni P, King JA (2007). Imaging unconditioned fear response with manganese-enhanced MRI (MEMRI). Neuroimage.

[13] Howles GP, Qi Y, Johnson GA (2010). Ultrasonic disruption of the blood-brain barrier enables *in vivo* functional mapping of the mouse barrel field cortex with manganese-enhanced MRI. Neuroimage.

[14] Pautler RG, Koretsky AP (2002). Tracing odor-induced activation in the olfactory bulbs of mice using manganese-enhanced magnetic resonance imaging. Neuroimage.

[15] Weng J-C, Chen J-H, Yang P-F, Tseng W-YI (2007). Functional mapping of rat barrel activation following whisker stimulation using activity-induced manganese-dependent contrast. Neuroimage.

[16] Lu H (2008). Real-time animal functional magnetic resonance imaging and its application to neuropharmacological studies. Magn. Reson. Imaging.

[17] Sun N (2006). Dynamic changes in orbitofrontal neuronal activity in rats during opiate administration and withdrawal. Neuroscience.

[18] Kuo Y-T, Herlihy AH, So P-W, Bell JD (2006). Manganese-enhanced magnetic resonance imaging (MEMRI) without compromise of the blood-brain barrier detects hypothalamic neuronal activity *in vivo*. NMR Biomed..

[19] Kuo Y-T, Herlihy AH, So P-W, Bhakoo KK, Bell JD (2005). *In vivo* measurements of T1 relaxation times in mouse brain associated with different modes of systemic administration of manganese chloride. Journal of magnetic resonance imaging: JMRI.

[20] Kimura T (2007). Hyperphosphorylated tau in parahippocampal cortex impairs place learning in aged mice expressing wild-type human tau. The EMBO journal.

[21] Inui-Yamamoto C (2010). The brain mapping of the retrieval of conditioned taste aversion memory using manganese-enhanced magnetic resonance imaging in rats. Neuroscience.

[22] Fontaine SN (2017). Identification of changes in neuronal function as a consequence of aging and tauopathic neurodegeneration using a novel and sensitive magnetic resonance imaging approach. Neurobiol. Aging.

[23] Perez PD (2013). *In vivo* functional brain mapping in a conditional mouse model of human tauopathy (tauP301L) reveals reduced neural activity in memory formation structures. Mol. Neurodegener..

[24] Tang X (2016). Spatial learning and memory impairments are associated with increased neuronal activity in 5XFAD mouse as measured by manganese-enhanced magnetic resonance imaging. Oncotarget.

[25] Brown MW, Aggleton JP (2001). Recognition memory: what are the roles of the perirhinal cortex and hippocampus?. Nature Reviews. Neuroscience.

[26] Casas C (2004). Massive CA1/2 neuronal loss with intraneuronal and N-terminal truncated Abeta42 accumulation in a novel Alzheimer transgenic model. Am. J. Pathol..

[27] Terleph, T. A. & Tremere, L. A. (eds R. Pinaud & L. A. Tremere) 1–10 (Springer Science 2006).

[28] Tischmeyer W, Grimm R (1999). Activation of immediate early genes and memory formation. Cell. Mol. Life Sci..

[29] Kumar-Singh S (2005). Dense-core plaques in Tg2576 and PSAPP mouse models of Alzheimer’s disease are centered on vessel walls. Am. J. Pathol..

[30] Cotel M-C, Jawhar S, Christensen DZ, Bayer TA, Wirths O (2012). Environmental enrichment fails to rescue working memory deficits, neuron loss, and neurogenesis in APP/PS1KI mice. Neurobiol. Aging.

[31] Wirths O, Breyhan H, Schafer S, Roth C, Bayer TA (2007). Deficits in working memory and motor performance in the APP/PS1ki mouse model for Alzheimer’s disease. Neurobiol. Aging.

[32] Silva AC, Lee JH, Aoki I, Koretsky AP (2004). Manganese-enhanced magnetic resonance imaging (MEMRI): methodological and practical considerations. NMR Biomed..

[33] Montagne A, Zhao Z, Zlokovic BV (2017). Alzheimer’s disease: A matter of blood-brain barrier dysfunction?. The Journal of experimental medicine.

[34] Berkowitz BA (2013). MRI biomarkers for evaluation of treatment efficacy in preclinical diabetic retinopathy. Expert Opin. Med. Diagn..

[35] Breyhan H (2009). APP/PS1KI bigenic mice develop early synaptic deficits and hippocampus atrophy. Acta Neuropathol..

[36] Benice TS, Raber J (2008). Object recognition analysis in mice using nose-point digital video tracking. J. Neurosci. Methods.

[37] Yu X, Wadghiri YZ, Sanes DH, Turnbull DH (2005). *In vivo* auditory brain mapping in mice with Mn-enhanced MRI. Nat. Neurosci..

[38] Chuang K-H, Koretsky AP, Sotak CH (2009). Temporal changes in the T1 and T2 relaxation rates (DeltaR1 and DeltaR2) in the rat brain are consistent with the tissue-clearance rates of elemental manganese. Magn. Reson. Med..

[39] Schneider CA, Rasband WS, Eliceiri KW (2012). NIH Image to ImageJ: 25 years of image analysis. Nat Methods.

